# Comparative Study of Microstructure and Phase Composition of Amorphous–Nanocrystalline Fe-Based Composite Material Produced by Laser Powder Bed Fusion in Argon and Helium Atmosphere

**DOI:** 10.3390/ma17102343

**Published:** 2024-05-15

**Authors:** Danil Erutin, Anatoliy Popovich, Vadim Sufiiarov

**Affiliations:** Institute of Mechanical Engineering, Materials, and Transport, Peter the Great St. Petersburg Polytechnic University, 195251 St. Petersburg, Russia

**Keywords:** laser powder bed fusion, bulk metallic glass, inert gas, additive manufacturing, amorphous phase

## Abstract

Laser powder bed fusion (LPBF) is a prospective and promising technique of additive manufacturing of which there is a growing interest for the development and production of Fe-based bulk metallic glasses and amorphous–nanocrystalline composites. Many factors affect the quality and properties of the resulting material, and these factors are being actively investigated by many researchers, however, the factor of the inert gas atmosphere used in the process remains virtually unexplored for Fe-based metallic glasses and composites at this time. Here, we present the results of producing amorphous–nanocrystalline composites from amorphous Fe-based powder via LPBF using argon and helium atmospheres. The analysis of the microstructures and phase compositions demonstrated that using helium as an inert gas in the LPBF resulted in a nearly three-fold increase in the amorphization degree of the material. Additionally, it had a beneficial impact on phase composition and structure in a heat-affected zone. The received results may help to develop approaches to control and improve the structural-phase state of amorphous–nanocrystalline compositional materials obtained via LPBF.

## 1. Introduction

LPBF has emerged as a promising additive manufacturing technique for producing complex metallic parts with unique properties. In recent years, there has been a growing interest in developing Fe-based bulk metallic glasses (BMGs) using LPBF, due to their potential for high strength, ductility, resistance to corrosion, and soft-magnetic properties [1]. Amorphous materials are defined by a disordered arrangement of atoms and the lack of long-range order. This state is achieved through the rapid cooling of the melt, which preserves the original positions of atoms. The resulting structural state is characterized by the absence of crystal structure defects, which can cause anisotropy in the functional and structural properties. Amorphous materials have unique structural properties that increase strength, magnetic softness, and corrosion resistance. Amorphous alloys, especially those based on iron, are widely used as magnetically conductive elements in electrical engineering due to their high magnetic softness [2,3,4,5,6,7]. Industrial production of such materials involves casting into water-cooled copper molds, melt spinning, and thermoplastic molding. However, these classical production technologies have limitations on the geometric shape and size of products, which restricts the range of products that can be produced from amorphous alloys. These technologies do not achieve sufficient cooling rates to fully amorphize the product.

However, the quality of the final product is heavily influenced by the initial powder characteristics [8,9,10] and processing conditions, including process parameters [11,12,13,14,15,16,17,18,19,20,21,22,23], scanning strategy [16,17,18,19,20], and protective atmosphere used [24,25,26]. Several studies have investigated the effect of protective atmospheres on the properties of LPBF-produced materials.

For example, Donghua Dai and colleagues studied the effect of metal vaporization behavior on the keyhole-mode surface morphology of AlSi10Mg matrix composites obtained via LPBF using different protective atmospheres [24]. They found that the choice of protective atmosphere can significantly affect the surface roughness and porosity of the resulting material. Authors reported that bulk density of the AlSi10Mg matrix composite material, reinforced with TiC nanoparticles, differed significantly under argon (98.5%), nitrogen (91%) and helium (85%) atmospheres.

Socona Traore and colleagues, in turn, investigated the influence of gas atmosphere (Ar or He) on the LPBF of a Ni-based alloy [25]. They discovered that the use of a helium atmosphere resulted in an increased volume of melted material under the working substrate. On the other hand, the denuded width of the single bead was found to have decreased under helium atmosphere, and a spattering phenomenon was also observed, which was found to be diminished in relation to argon atmosphere. Observed differences in the mentioned material properties were attributed to the differences in thermal conductivity and mass transfer between the two gases. 

In this study, we investigate the effect of changing the protective gas from argon to helium on the bulk density, phase composition, amorphous phase content, and microstructural features of the Fe-based amorphous–nanocrystalline composite material obtained via LPBF. Our goal was to evaluate the impact of the protective atmosphere on the resulting material properties and to identify the optimal atmosphere for producing high-quality Fe-based amorphous–nanocrystalline composites using LPBF. The significance of this research lies in its ability to provide insights into the optimization of the processing conditions for producing high-performance Fe-based BMGs. The findings of this study could contribute to the development of novel materials with improved mechanical, thermal, and magnetic properties, which could have far-reaching implications for various industries, such as aerospace, automotive, and energy. Moreover, the understanding gained from this study could help to establish the use of LBPF as a reliable method for fabricating large-scale, complex parts with tailored properties, thereby expanding the range of applications for additive manufacturing technology.

## 2. Materials and Methods

**Figure 1:**
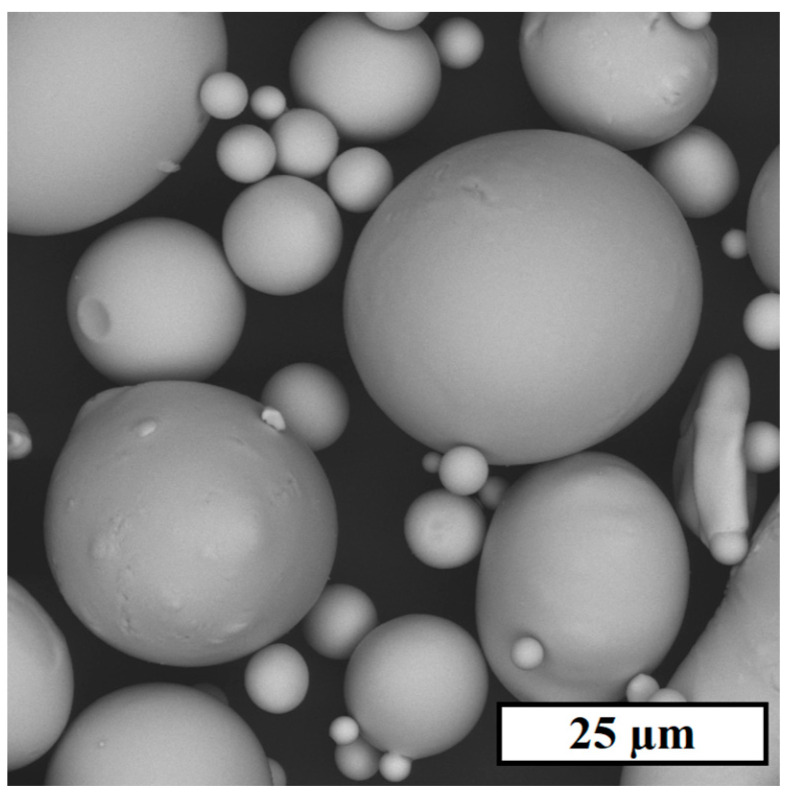
SEM image of AP-02 powder.

An initial powder of AP-02 (FeC_0.8_Si_6.63_Cr_2.40_B_2.28_) was used for experimental part production, consisting mostly of spherical particles (Figure 1) with a mean size of 21.2 µm. The powder seems to have a good spreading ability, which is vital to form a thin and flat powder layer during LPBF. Also, it should be noted that the powder’s ability to form an acceptable packing density during layer formation is consistent with 59% of the ratio of its apparent (4.01 g/cm^3^) and skeletal (6.80 g/cm^3^) densities.

Experimental samples of elliptical prism geometry (15 mm × 30 mm × 5 mm) were built in two experimental sessions using two different shielding gas atmospheres of argon (Ar-SLM) and helium (He-SLM) within the framework of 3DLAM Mini LPBF system (3DLam, St. Petersburg, Russia), equipped with a 300 W continuous wave Ytterbium fiber laser (wavelength of 1070 ± 10 nm) with Gaussian power distribution of the beam. Samples were then fabricated with a volume energy density of 78.95 J/mm^3^ and powder layer thickness of 20 µm using the hatch filling scanning strategy with a layer rotation angle of 67°. Track scanning was performed sequentially one by one. 

The microstructure and density of the experimental materials were examined using an optical microscope. To conduct the research, samples were pressed into the resin. After the specimens were obtained, grinding and polishing were performed to finish the surface. Image analysis was performed, based on 5 images of the longitudinal section for each sample, with the specialized ImageJ software version 1.54 [26]. Microstructural characterization of the samples was performed with the Tescan Mira 3M scanning electron microscope in secondary electron mode (Brno, Czech Republic). To reveal the microstructure, samples were etched in 25% solution of nitric acid in water.

The amorphous phase crystallization enthalpies of the initial powder and obtained samples were studied with differential scanning calorimeter (DSC) equipped with an automatic sampler, baseline alignment technology, and cooling system. Studied specimens were heated to a temperature of 1000 °C with a heating rate of 20 °C/min. Thereafter, the cooled samples were heated again to the same temperature. All the DSC heating operations were performed in an argon atmosphere to prevent the studied material from oxidation. 

**Figure 2:**
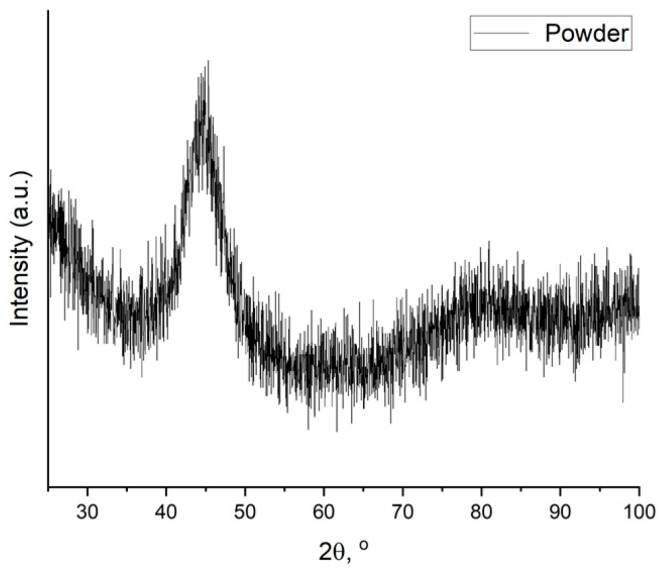
X-ray diffraction pattern of the powder material.

Phase analysis of the powder and samples was performed with X-ray diffractometer using Cu Kα (1/4 1.5418 Å) irradiation. The diffraction pattern of the initial powder is presented in Figure 2. The XRD halo indicates that initial powder material is completely amorphous.

## 3. Results

**Figure 3:**
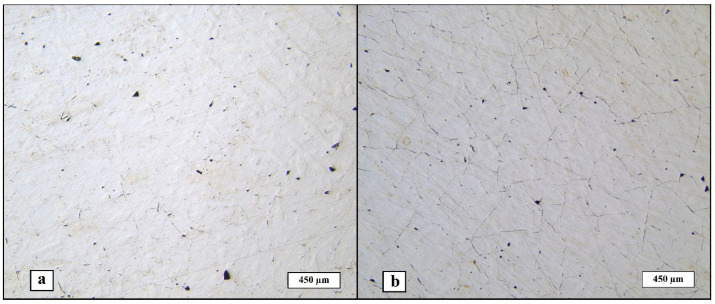
Macrostructure of the material obtained: (**a**) Ar-SLM, (**b**) He-SLM.

Figure 3 shows the structure of the manufactured materials. Relative density percentage was calculated for both, and the mean values are 98.66% ± 0.06 for Ar-SLM and 96.45% ± 0.04 for He-SLM.

Both micro-cracks and pores are present in the obtained structures. Micro-cracking is a typical effect of the internal stress induced by the phase transitions and non-uniform cyclic heating during the laser powder bed fusion process on the resulting material’s structure [27,28]. It can be seen that the micro-cracking phenomenon is presented largely in He-SLM, which can be attributed to the numerically predicted melt pool depth fluctuations during the LPBF under helium atmosphere and flow [24]. The difference between the melt pool depth of the closely located laser beam hatches may result in a non-uniform distribution of the internal stress field caused by heating and subsequent cooling of the layer part, which, in turn, creates opportunities for crack initiation and propagation. However, micro-cracks are presented in Ar-SLM as well but, considering its structure, it allows to conclude that continuous or quasi-continuous net of micro-cracks is absent as opposed to the He-SLM structure. Another possible origin of the increased cracking of He-SLM is an increased denudation efficiency (at approximately equal denudation zone widths in helium and argon atmospheres) of a single laser beam track under helium atmosphere reported in [25] for a Gaussian distributed laser beam, denoting the share of missing powder particles of those in the denudation zone. The denudation effect lies in dragging the particles from a side of the laser beam path to the melt pool during scanning, decreasing the quantity of particles located on a side of the melted track. The denudation is common for the metal LBPF process and is also a consequence of an intensive evaporation by the entrainment flow of ambient gas induced by a vapor jet. The entrainment flow arises due to the Bernoulli effect and is directed toward the vapor jet, which leads to the movement of powder particles toward the melt pool [29]. Hence, increased denudation efficiency might lead to a decrease in the adjacent tracks overlapping due to the lack of powder, which is detrimental for the geometrical uniformity of the melted layer. Such specialty of interaction between powder particles and the laser beam may also lead to pre-melting of a side volume of particles, not being involved in the melt pool itself. Considering such an interaction allows us to assume that value of the pre-melted side volume of the material depends on the width of the denudation zone and denudation efficiency; hence, wider and more “efficient” denudation zones promote more pre-melted particles on a side of the melt pool. In that case, each next laser track will interact not only with the powder particles, but also with mentioned pre-melted volume. Therefore, the melt pool should be stabilized by the substitution of the portion of the particles’ volume involved in the melt pool by a pre-melted material, in which the geometry is already constituted, in contrast with the array of single particles. On the other hand, such a material laying separate from the laser track way may consume slightly more thermal energy produced by laser beam in comparison to an array of particles. Therefore, such pre-melting effects create two factors, partially determining the quality of the resulting material, in terms of melt pool stabilization and a minor lack of thermal energy. In view of the described theoretical approach and according to [25], it can be concluded that a decreased denudation zone efficiency during LPBF in argon flow and atmosphere is another circumstance resulting in a slightly increased relative density of the obtained material as opposed to the material obtained with helium shielding gas. Thereafter, the considered roots of the increased micro-cracking of the material obtained using helium as a shielding gas are based on the melt pool stability and melted layer geometrical uniformity during LPBF.

**Figure 4:**
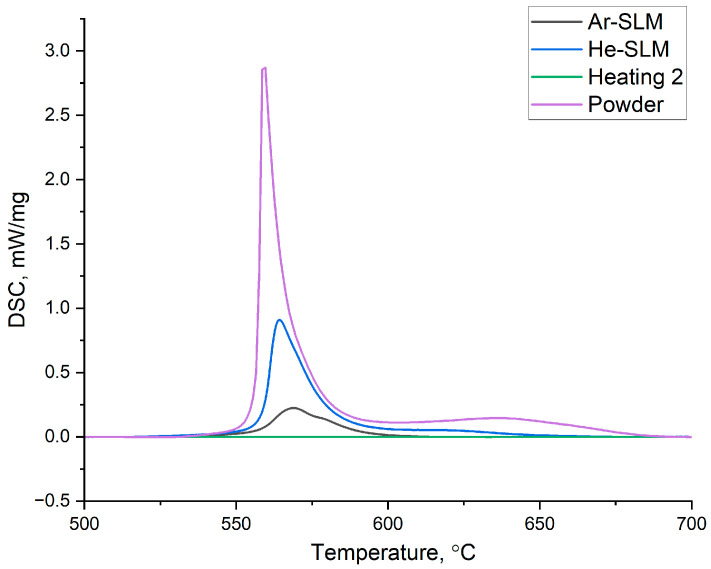
Amorphous phase crystallization peaks on the differential scanning calorimetry heating curves for the powder and experimental materials.

Differential scanning calorimetry investigation results are presented in Figure 4. Amorphous phase crystallization enthalpies were calculated and amounted to 16.39 J/g and 44.87 J/g for Ar-SLM and He-SLM, respectively.

For the fully amorphous AP-02 powder, the amorphous phase crystallization enthalpy is 110.25 J/g. Hence, it can be concluded that the amorphization degree of the Ar-SLM is 14.85% and that of He-SLM is 40.69%. Amorphization degree and amorphous phase distribution in the amorphous–nanocrystalline composite are very important as these characteristics have an impact on the resulting mechanical and functional properties of the material. Formation of the amorphous phase under conditions of laser powder bed fusion is determined by the cooling rate of the molten part of the powder layer and its thermal exposure on the already solidified tracks, resulting in the formation of a heat affection zone (HAZ) in the final structure, under the condition of constancy of the material’s glass-forming ability. Considering the given gas atmosphere in the working chamber, the cooling rate of the single laser beam track is determined by laser power, scanning speed, and thickness of the powder layer. To become amorphous, an elementary volume of the molten metal must be cooled with the rate exceeding the critical value under the glass transition temperature. However, during LPBF, an almost inevitable crystallization occurs due to the heating of the obtained amorphous phase, caused by a meltdown of the adjacent material volume. HAZ, in which the crystallization occurs, is geometrically determined not only by the mentioned basic parameters, but also by the performed scanning strategy, melting sequence, and hatch distance. The difference between the amorphization degrees of the obtained materials should be considered in terms of the following factors: cooling rate and HAZ configuration. A numerical simulation of the cooling rate of the melt pool reported in [30] for Ti-based alloy showed a significant difference in the heat transfer coefficient between molten metal and gas in the context of using helium and argon. The obtained value of the coefficient was approximately four times higher for helium, and the increased heat dissipation from the melt pool surface in the helium atmosphere was confirmed by microstructural analysis, which showed the grain refinement in the microstructure of He-SLM relative to Ar-SLM. Therefore, it can be assumed that the use of helium as a shielding gas during laser powder bed fusion allows us to amplify the cooling rate of the melt pool by an increased heat dissipation to the gas.

**Figure 5:**
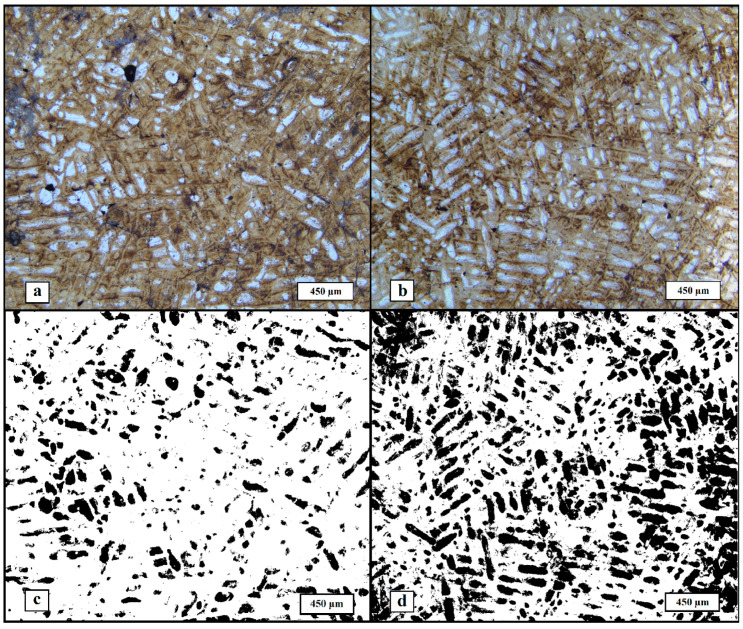
Microstructure of the materials, images obtained with optical microscopy: (**a**) microstructure of Ar-SLM, (**b**) microstructure of He-SLM, (**c**) amorphous phase in Ar-SLM (dark regions), and (**d**) amorphous phase in He-SLM (dark regions).

Microstructural analysis was performed on the etched samples with optical microscopy (Figure 5). In general, two types of regions are visible on the images, namely dark etched regions of the crystalline phase and white amorphous phase regions. As expected, the formed structure can be classified as compositional; however, the structural component ratio of the materials is different. Based on the images, it can be said that the microstructure of He-SLM contains noticeably more amorphous phase relative to Ar-SLM, which qualitatively confirms the obtained DSC data. To measure the share of the amorphous phase, contrasting was performed and the area of the dark (contrasted amorphous) regions was calculated. The results of the DSC data are quantitatively demonstrated by mean amorphous region area values of 14.56% and 34.77% for materials obtained in argon and helium, respectively. Discrepancies in the values of amorphization degree calculated with DSC and its estimation obtained from image analysis subsists on the fact that DSC analysis provides a volume-based value, while image analysis only allows us to obtain a surface-based value.

**Figure 6:**
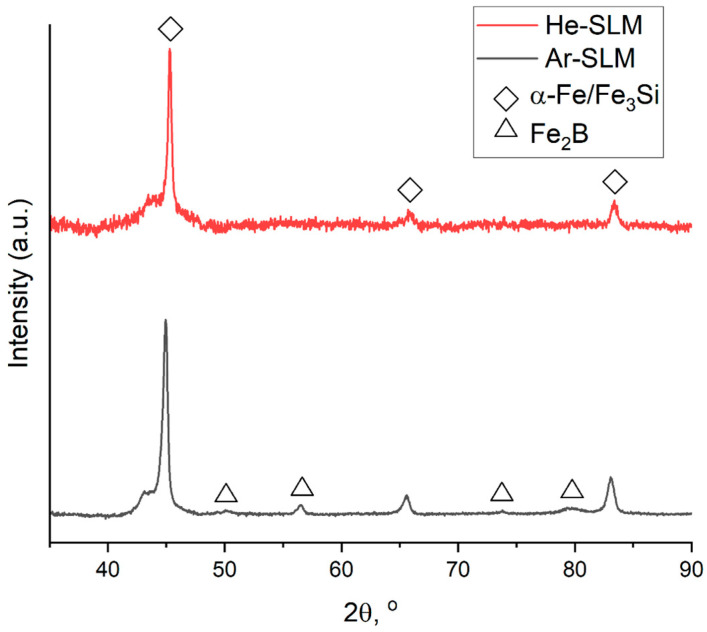
X-ray diffraction patterns of the materials.

X-ray diffraction patterns for the materials (Figure 6) shows that the crystalline phase identified as α-Fe(Si) exists in both obtained structures. Considering other research of LPBF-ed Fe-based alloys [9,14,19,20] allows us to assume the coexistence of α-Fe(Si) and ordered Fe_3_Si, which is characterized by a similar crystalline lattice with almost equal parameters relative to α-Fe(Si), so it is not visible on the diffraction pattern. But there is a major difference in the phase compositions of the materials, appearing in the fact that peaks related to iron boride (Fe_2_B) are absent on the pattern of He-SLM. To determine the cause of such a difference, the origins of the presented crystalline phases should be clarified. Research provided by Zrodowski and his colleagues [19] was dedicated to LPBF experiments with Fe-based amorphous powder, and within the scope of this study, it was pointed out that the ordered solution (Fe_3_Si) and iron boride (Fe_2_B) are formed through devitrification of the amorphous phase. Moreover, iron boride (Fe_2_B) is formed from the metastable boride phase of Fe_3_B during its heating. Based on these findings, the ordered solution and iron boride should be observed in HAZ, and the α-Fe(Si) solution should be observed in the amorphous zone as a phase forming through insufficient cooling of the parts of the melt pool.

**Figure 7:**
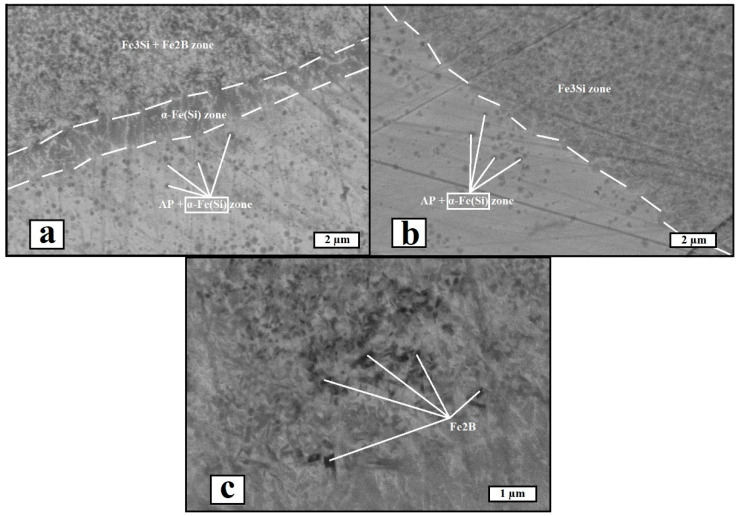
SEM images of the materials (SE mode, AP—amorphous phase): (**a**) image of interphase zone in Ar-SLM, (**b**) image of interphase zone in He-SLM, and (**c**) image of iron boride (Fe_2_B) precipitations in Ar-SLM.

Scanning electron microscopy was used to investigate the microstructure of the materials. Figure 7 shows the microstructural images obtained in the secondary electron mode. An interphase boundary zone was observed for both materials, and it was testified that iron boride precipitations are absent in the vicinity of the phase boundary between the amorphous phase and ordered solid solution in He-SLM, while in the microstructure of Ar-SLM, boride precipitations were observed within the ordered solution zones. Therefore, the X-Ray diffraction data are consistent with the observed microstructure. A common feature of the microstructure for both materials is the presence of α-Fe(Si) precipitations within the amorphous regions. Another major difference between the microstructural condition of the materials is the existence of an intermediate α-Fe(Si) zone between amorphous region and ordered solution zone in Ar-SLM, which cannot be said about He-SLM.

Considering the differences of the heat transfer coefficients between molten metal and gas in the context of argon and helium atmospheres allows us to find a correlation between the cooling rate of both the liquid and solid material states and the phases presented in the interphase zone. Apparently, iron boride formation within the structure of He-SLM was inhibited, along with the α-Fe(Si) interlayer. In view of the mechanism of iron boride formation, its absence implies the lack of heating within the solidified material surrounding the melt pool. Under the condition of the same energy input during melting under argon and helium, the lack of a change in temperature in HAZ may be attributed to increased heat dissipation from the melt pool and surrounding volume of the material. Considering HAZ in He-SLM, forming the temperature field leads to a formation of the ordered solution, Fe_3_Si, but the temperature of the field periphery cannot reach the values required to subsequently form Fe_3_B and Fe_2_B. As for the α-Fe(Si) interlayer, such structural speciality might form in conditions of melting and subsequent insufficient cooling of the pre-melted material.

## 4. Conclusions

LPBF experiments performed using argon and helium as a protective gas showed that the use of helium affects the bulk density, phase composition, amorphization degree, and microstructure of the obtained amorphous–nanocrystalline Fe-based composite material. During the study, it was found that laser powder bed fusion in helium atmosphere contributes to a slightly increased cracking of the material, resulting in decreased relative density as compared to the material obtained with argon gas, which may be attributed to a reduced melt pool stability. However, the use of helium significantly affected the phase composition and microstructure of the resulting material. The amorphization degree of the helium-built material calculated from DSC turned out to be almost three times higher than that of the argon-built one, which was confirmed by analysis of the microstructure obtained with optical microscopy. The microstructures of the interphase zones of the materials were studied using SEM, and it was found that the α-Fe(Si) interlayer zone between the ordered solution zone and the amorphous region is absent in helium-built material, as well as iron boride Fe_2_B precipitations, which were found only in the ordered solution zone of argon-built material. Hence, the use of helium significantly improved the phase composition of the resulting material.

The obtained results can be used in the development of technology for manufacturing products with bulk metallic glasses or amorphous–nanocrystalline microstructures, such as components of next-generation electromagnetic motors, magnetic shields, etc.

## Data Availability

Data are contained within the article.
